# Neurally adjusted ventilatory assist as a weaning mode for adults with invasive mechanical ventilation: a systematic review and meta-analysis

**DOI:** 10.1186/s13054-021-03644-z

**Published:** 2021-06-29

**Authors:** Xueyan Yuan, Xinxing Lu, Yali Chao, Jennifer Beck, Christer Sinderby, Jianfeng Xie, Yi Yang, Haibo Qiu, Ling Liu

**Affiliations:** 1 Jiangsu Provincial Key Laboratory of Critical Care Medicine, Department of Critical Care Medicine, Zhongda Hospital, School of Medicine, Southeast University, Nanjing, 210009 Jiangsu China; 2 Department of Pediatrics, University of Toronto, Toronto, Canada; 3 Department of Medicine and Interdepartmental Division of Critical Care Medicine, University of Toronto, Toronto, Canada; 4 Department of Critical Care, Keenan Research Centre for Biomedical Science of St. Michael’s Hospital, St. Michael’s Hospital, 30 Bond Street, Toronto, ON M5B1W8 Canada; 5 Institute for Biomedical Engineering and Science Technology (iBEST), Ryerson University and St-Michael’s Hospital, Toronto, Canada

**Keywords:** Mechanical ventilation, Neurally adjusted ventilatory assist, Weaning success, Patient–ventilator asynchrony

## Abstract

**Background:**

Prolonged ventilatory support is associated with poor clinical outcomes. Partial support modes, especially pressure support ventilation, are frequently used in clinical practice but are associated with patient–ventilation asynchrony and deliver fixed levels of assist. Neurally adjusted ventilatory assist (NAVA), a mode of partial ventilatory assist that reduces patient–ventilator asynchrony, may be an alternative for weaning. However, the effects of NAVA on weaning outcomes in clinical practice are unclear.

**Methods:**

We searched PubMed, Embase, Medline, and Cochrane Library from 2007 to December 2020. Randomized controlled trials and crossover trials that compared NAVA and other modes were identified in this study. The primary outcome was weaning success which was defined as the absence of ventilatory support for more than 48 h. Summary estimates of effect using odds ratio (OR) for dichotomous outcomes and mean difference (MD) for continuous outcomes with accompanying 95% confidence interval (CI) were expressed.

**Results:**

Seven studies (*n* = 693 patients) were included. Regarding the primary outcome, patients weaned with NAVA had a higher success rate compared with other partial support modes (OR = 1.93; 95% CI 1.12 to 3.32; *P* = 0.02). For the secondary outcomes, NAVA may reduce duration of mechanical ventilation (MD = − 2.63; 95% CI − 4.22 to − 1.03; *P* = 0.001) and hospital mortality (OR = 0.58; 95% CI 0.40 to 0.84; *P* = 0.004) and prolongs ventilator-free days (MD = 3.48; 95% CI 0.97 to 6.00; *P* = 0.007) when compared with other modes.

**Conclusions:**

Our study suggests that the NAVA mode may improve the rate of weaning success compared with other partial support modes for difficult to wean patients.

**Supplementary Information:**

The online version contains supplementary material available at 10.1186/s13054-021-03644-z.

## Background

Mechanical ventilation (MV) remains a lifesaving intervention for patients with respiratory failure of different etiologies, and 20–60% of patients admitted to the intensive care unit (ICU) required ventilatory support [1, 2]. While invasive MV maintains the airway, its use over a prolonged period is associated with adverse clinical complications and outcomes [3, 4]. In turn, these complications contribute to weaning failure, duration of intubation, and ICU mortality. Consequently, optimizing strategies for weaning and minimizing the duration of invasive MV have been an important goal to reduce morbidity and mortality [5].

Weaning from MV is an essential and universal process for intubated patients in the ICU—it should be considered as early as possible in the course of MV. Weaning refers to the efficient process of withdrawing ventilator support, including discontinuation of MV and removal of the artificial airway [6]. The transition from controlled MV to spontaneous breathing can be achieved by modes of partial ventilatory assist and may minimize the adverse effects of diaphragm inactivity and excessive sedation [7–9]. Pressure support ventilation (PSV) is the most widely used mode of partial assistance. However, the imperfectly set and fixed level of inspiratory support in PSV may increase the risk of over- and/or under-assist ventilatory and may lead to diaphragm weakness or excessive diaphragm work [10]. Not to be ignored is the fact that PSV demonstrates patient–ventilator asynchrony (PVA), which occurs in approximately 25% of patients with invasive MV and is known to be associated with prolonged of duration of MV and increased mortality [11–14].

Neurally adjusted ventilatory assist (NAVA) is a mode of partial ventilatory assist in which the timing and intensity of the ventilatory assistance are determined by the electrical activity of the diaphragm (Edi) [15], a measurement of respiratory drive. Hence, NAVA delivers inspiratory pressure in proportion to the patient’s inspiratory effort. Previous studies have shown that NAVA can reduce PVA and lung overdistension [16, 17]. In several studies, NAVA has shown physiological benefits compared to PSV with respect to preferential recruitment of the dependent part of lung [18], improved gas exchange and sleep [19, 20], and less sedation requirements [21]. These findings suggest that NAVA—being more physiological—could optimize weaning. However, the evidence on weaning and clinically relevant outcomes for NAVA is limited.

Therefore, we conducted a systematic review and meta-analysis to assess the effectiveness of NAVA for intubated patients with respiratory failure by measuring weaning success and other clinically relevant outcomes.

## Methods

We adhered to the Preferred Reporting Items for Systematic Reviews and Meta-analyses (PRISMA) statement for performing the systematic review and meta-analysis [22]. Our study protocol was registered at PROSPERO with the registration number CRD42021225997.

### Criteria for considering studies for this review

We included all randomized controlled trials (RCTs) including those with crossover design. Relevant conference abstracts were considered for inclusion after contacting the authors. Studies were eligible if they (i) included adults (aged 18 years or older) with respiratory failure from various etiologies who received invasive MV for least 24 h, (ii) compared NAVA with partial support modes (PSV, assist/control (A/C), or pressure-regulated volume control (PRVC)), and (iii) included patients who were undergoing weaning trials for liberation from MV. We excluded studies that extubated patients directly to non-invasive ventilation.

The primary outcome was weaning success, which was defined as the absence of the requirement for ventilatory support, without reintubation, a cardiac arrest event, or mortality within 48 h after extubation or withdrawal. The secondary outcomes included duration of MV, ventilator-free days at day 28 (VFDs), ICU mortality, hospital mortality, length of hospital stay (hospital LOS), adverse events, and tracheostomy.

### Search strategy

We conducted an electronic search of PubMed, Embase, Medline, and Cochrane Library for all relevant studies from 2007 to December 2020. We restricted the articles to those published in English. The details of the search strategies used for each database are presented in the Additional file 1: Table S1. In addition, we manually retrieved the bibliographies of all relevant studies and reviews to identify potentially eligible studies.

### Data collection and analysis

#### Selection of studies

We merged the search results and removed the duplicate records of the same study. Two authors (XY and XL) independently reviewed the titles and abstracts of all studies identified by the initial search strategy for potential eligibility and retrieved the potentially relevant studies for full-text review. Data were independently extracted by two authors (XY and YC) using a standardized data collection form. Disagreements were resolved through consensus with a third author (JX).

#### Assessment of risk of bias in included studies

Two authors (XL and YC) assessed the risk of bias using the Cochrane Collaboration’s tool for RCTs [23] and the checklist proposed by Ding et al. [24] for crossover studies, both independently and in duplicate. Specifically, the methodological quality of crossover studies was assessed with appropriate crossover design, carryover effect, unbiased data, randomized sequence generation, allocation concealment, blinding, incomplete outcome data, selective outcome reporting, and other bias. The overall risk of bias was considered to be high if any individual category was high. The overall certainty of evidence for each outcome was assessed by the Grading of Recommendations Assessment, Development and Evaluation (GRADE) framework [25]. Disagreements were resolved through a consensus with a third author (JX).

#### Assessment of heterogeneity

Heterogeneity was evaluated using the Cochran’s Q test, a Chi-square test, with a threshold P value of less than 0.10 [26]. The impact of heterogeneity on outcomes was assessed using *I*^2^ statistic [27]. *I*^2^ greater than or equal to 50% was considered as statistically significant heterogeneity.

#### Assessment of reporting biases

The presence of publication bias on the primary outcome was assessed using funnel plots and Egger’s test [28].

#### Data synthesis

For dichotomous variables, the estimated effects were pooled with Mantel–Haenszel method and expressed with the odds ratio (OR) with 95% confidence interval (CI). For the continuous variables, the estimated effects were pooled with the inverse variance method and expressed with the mean difference (MD) with 95% CI. To statistically combine the data from the included studies, the median along with the 25% and 75% percentiles was transformed into means and standard deviation using the method proposed by Liu et al. [29]. The choice between fixed-effect or random-effect models was based on statistical heterogeneity. If *P* < 0.10 with the Chi-square test or *I*^2^ > 50%, a random-effects model was used to pool data; otherwise, the fixed-effect model was used.

#### Subgroup analysis

For the primary outcome, we conducted subgroup analysis by the weaning state (i.e., simple weaning, difficult weaning, prolonged weaning).

#### Trial sequential analysis (TSA)

We used TSA to identify the risk of both type 1 and type 2 error due to sparse data and repetitive testing of accumulating data for primary outcome in our meta-analysis [30]. The Lan-DeMets approach was used to estimate the required information size and construct O’Brien–Fleming monitoring boundaries. We defined a statistical significance level of 5%, a power of 80%, and a relative risk reduction of 35%. TSA was conducted by Trial Sequential Analysis software (version 0.9.5.10 Beta 2; Copenhagen Trial Unit, Copenhagen, Denmark).

All statistical analysis was performed using RevMan 5.3 (The Nordic Cochrane Centre, The Cochrane Collaboration, 2014). *P* < 0.05 was considered statistically significant.

## Results

### Results of the search

**Fig. 1:**
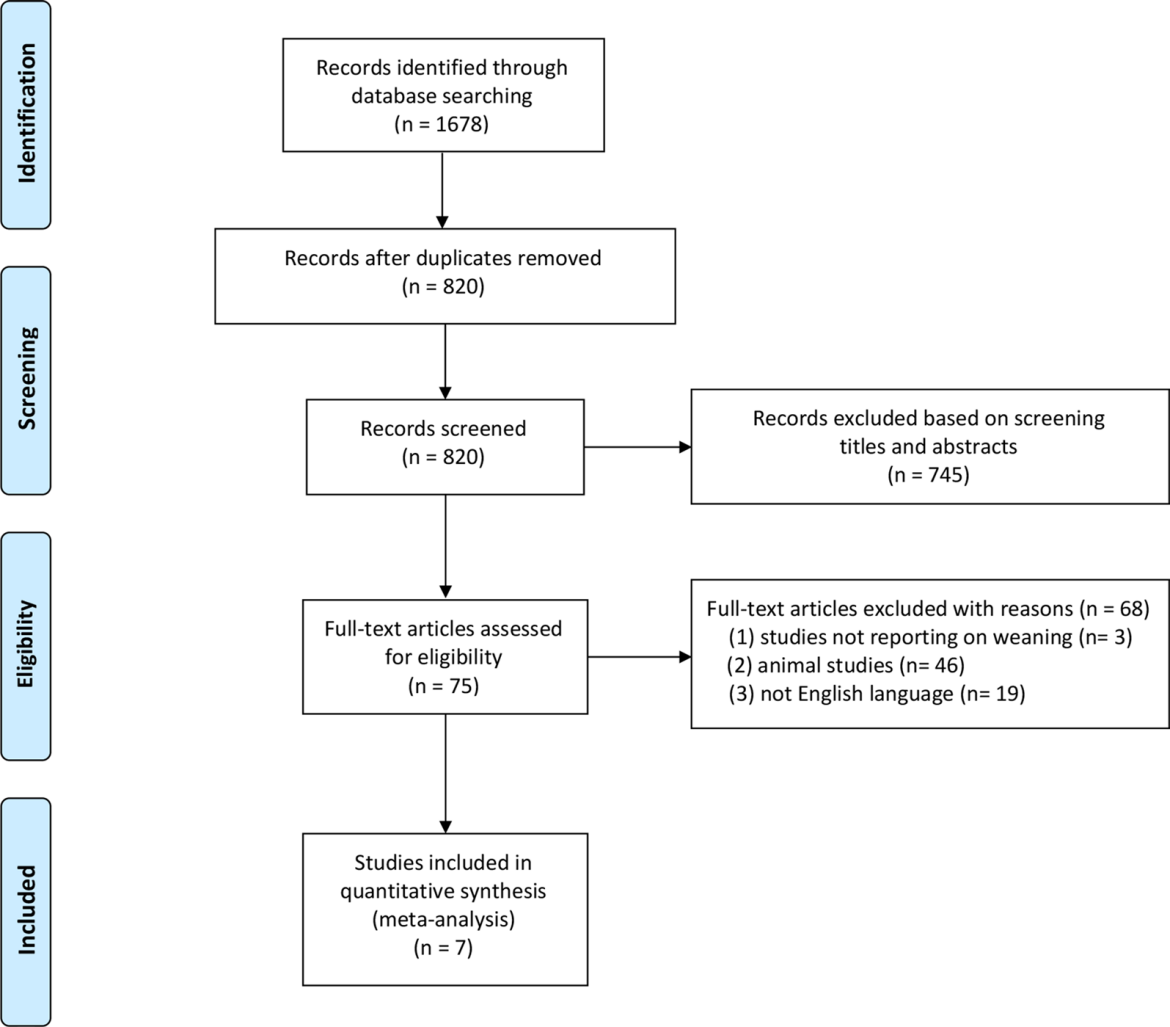
Flowchart of the selection process for the included studies

We identified 1678 records in accordance with the search strategy and retrieved the full text of 75 studies for possible eligibility. The flowchart of our search process is presented in Fig. 1. Of the 75 studies, 7 studies met all inclusion criteria and were included in the final quantitative synthesis [31–37]. The seven included studies comprised a total of 693 patients. One of them was a published conference abstract [31]. Of note, 4 of the 7 studies were included in the meta-analysis of weaning success [31, 33–35].

### Included studies

Study characteristics and patient characteristics are summarized in Tables 1 and 2. All the included studies were published between 2016 and 2020. Five studies were single-center studies [31–33, 35, 37] and 2 were multi-center studies [34, 36]. Among the seven studies, 6 RCTs and 1 crossover trial were identified. Assessment of the risk of bias in included studies is shown in Additional file 1: Figs. S1 and S2. The overall quality of these studies was low–moderate. Blinding of participants and personnel was impossible owing to the essence of study design, but the blinding of outcome assessment was applicable [32]. According to the difficulty and length of weaning process, we divided patients of the included studies into three categories, namely simple weaning, difficult weaning, and prolonged weaning [6]. Two studies included patients who were classified into difficult weaning [31, 35]. COPD patients in one study were assigned to the prolonged weaning group [37]. The remaining four studies included patients with more than one weaning category [32–34, 36].

**Table 1:** Characteristics of the included studies and patients

Authors (year)	Country	Study type	Settings	Inclusion criteria	Control strategy		Cause of invasive MV	Outcomes
					Mode	Support level (cmH_2_O)		
Demoule 2016	France	Multi-center RCT	ICU	Endotracheal MV more than 24 h	PSV	PS level: set to obtain a tidal volume of 6–8 ml/kg predicted body weight PEEP: set PEEP according to local guidelines	de novo hypoxemic RF acute cardiogenic pulmonary edema acute-on-chronic RF	Primary outcome: Proportion of patients with successful partial ventilator support Secondary outcomes: duration of MV; VFDs; hospital /ICU LOS
Kuo 2016	Taiwan, China	Single-center RCT	RCC	MV via intubation or tracheotomy, > 21 days	PSV	PS level: 8 PEEP: 5	COPD	Primary outcome: weaning outcome Secondary outcomes: length of MV, RCC/hospital LOS; hospital, and 90-day mortality
Ferreira 2017	Brazil	Single-center randomized crossover trial	ICU	MV more than 48 h	PSV	PS level: 5 PEEP: 5	COPD, trauma, sepsis, cardiac arrest, coma, metabolic acidosis, pneumonia, drowning, pleural effusion, cardiac failure	Outcome of SBT and extubations; asynchrony index; weaning success; length of MV; mortality; PaO2/FiO2 index
Fakher 2019	Egypt	Single-center RCT	ICU	Invasively ventilated with predictive criteria of difficult weaning	PSV	PS level: set to obtain a tidal volume of 5–8 ml/kg predicted body weight PEEP: NA	N/a	Weaning success; length of MV; length of stay; mortality; PaO2/FiO2 index
Hadfield 2020	UK	Multi-center RCT	ICU	Invasive MV with the risk factors for prolonged MV	PSV	PS level: set to obtain a tidal volume of 6–8 ml/kg predicted body weight PEEP: N/a	COPD, heart failure, ARDS	Primary outcome: the proportion of MV time^a^ Secondary outcomes: VFDs; duration of MV; ICU/hospital mortality; ICU/hospital LOS; VAP, pneumothoraxes, and unplanned extubation
Kacmarek 2020	Spain	Multi-center RCT	ICU	Intubated and mechanically ventilated for ≤ 5 days but expected to be ventilated for ≥ 72 h	PSV, A/C, PRVC	N/a	Pneumonia, sepsis, post-surgical, COPD, acute pancreatitis, aspiration, poisoning, trauma, heart failure, other	Primary outcome: VFDs Secondary outcomes: ICU/hospital mortality; duration of MV; reintubation rate; pulmonary complications
Liu 2020	China	Single-center RCT	ICU	Invasive MV more than 24 h	PSV	PS level: set to obtain a VT of 6 to 8 ml/kg predicted body weight PEEP: to maintain Spo2 ≥ 90%	Pneumonia, sepsis, COPD, ACS/CHF, nervous system disease, surgery, trauma, other	Primary outcomes: duration of weaning Secondary outcomes: successful weaning; successful extubation; VFDs; duration of invasive MV; hospital/ICU LOS; 28-day mortality; PVA

NAVA, neurally adjusted ventilatory assist; MV, mechanical ventilation; RCT, randomized controlled trial; ICU, intensive care unit; PSV, pressure support ventilation; RF, respiratory failure; COPD, chronic obstructive pulmonary disease; ARDS, acute respiratory distress syndrome; ACS, acute coronary syndrome; CHF, chronic heart failure; A/C, assist/control; PRVC, pressure-regulated volume control; PEEP, positive end-expiratory pressure; VFDs, ventilator-free days; LOS, length-of-stay; SBT, spontaneous breathing trials; VAP, ventilator-associated pneumonia; PVA, patient–ventilator asynchrony

^a^This is the time spent in assigned mode from randomization to extubation, death, or D28

**Table 2:** Baseline characteristics of patients

Authors (year)	Sample size (n)	Age (years)	Man n, (%)	SOFA	APACHE II	PaO2/FiO2 at enrolment	Duration of MV (days)	PEEP (cm H_2_O)	PS (cm H_2_O)
*Demoule 2016*									
NAVA	62	66 (61, 77)	47 (76)	n/a	n/a	235 (185, 265)	4 (2, 8)^*^	6 (5, 8)	12 (10, 16)
Control	66	64 (53, 77)	39 (59)	n/a	n/a	227 (192, 286)	5 (3, 8)^*^	6 (5, 8)	12 (10, 14)
*Kuo 2016*									
NAVA	14	79.3 ± 15.5	11 (78.6)	3.7 ± 2.8	17.4 ± 4.1	n/a	26.9 ± 10.7^&^	n/a	n/a
Control	19	76.9 ± 9.3	13 (68.4)	4.3 ± 2.5	18.7 ± 5.0	n/a	27.1 ± 13.0^&^	n/a	n/a
*Ferreira 2017*^a^									
NAVA	17	n/a	n/a	n/a	n/a	n/a	6.2 ± 2.8^*^	6.0 (5.2, 8.0)	10.0 (8.0, 11.5)
Control	20	n/a	n/a	n/a	n/a	n/a			
Fakher 2019									
NAVA	15	n/a	n/a	n/a	n/a	n/a	n/a	n/a	n/a
Control	15	n/a	n/a	n/a	n/a	n/a	n/a	n/a	n/a
*Hadfield 2020*									
NAVA	39	66.7 (13.9)	26 (66.7)	8.0 (6.0–8.0)	20.5 (6.0)	227.0 (82.0)	1.7 (1.1, 3.1)^#^	8.9 (2.7)	n/a
Control	38	67.1 (12.9)	28 (73.7)	8.0 (5.5–10.0)	20.1 (6.1)	242.0 (83.0)	1.7 (0.7, 3.0)^#^	8.9 (2.8)	n/a
Kacmarek 2020									
NAVA	153	63.9 (15.4)	100 (65.4)	6.4 ± 3.1	16.1 ± 7	250 ± 87	2.4 ± 1.5^#^	8 ± 2	n/a
Control	153	64.7 (14.1)	101 (66)	6.8 ± 3.3	16.4 ± 7.2	244 ± 88	2.0 ± 1.5^#^	8 ± 3	n/a
*Liu 2020*									
NAVA	47	80 (65, 80)	30 (64)	n/a	22 (16, 26)	279 (229,322)	5.0 (2.6, 7.6)^*^	5 (5, 6)	8 (8, 10)
Control	52	75 (61, 80)	36 (69)	n/a	20 (17, 28)	271 (230, 349)	5.9 (3.0, 10.8)^*^	5 (5, 6)	8 (7, 10)

SOFA, sequential organ failure assessment; APACHE II, acute physiology and chronic health evaluation; MV, mechanical ventilation; PEEP, positive end-expiratory pressure; PS, pressure support; NAVA, neurally adjusted ventilatory assist

^a^All of 20 patients including in the study conducted by Ferreira et al. underwent both NAVA and other partial support modes

^*^Durations of MV measured before inclusion

^&^Durations of MV measured before randomization

^#^Duration of invasive MV measured before randomization

### Primary outcome

**Fig. 2:**
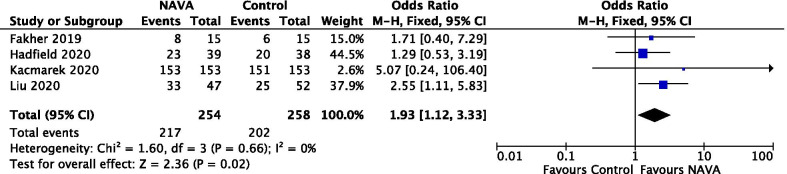
Forest plot for weaning success

A total of 4 studies, involving 512 patients, were included in the analysis [31, 33–35] for the primary outcome. Two other studies were excluded for lack of data on the weaning success [36, 37]. One crossover trial was excluded due to the potential risk for carryover effect [32]. The meta-analysis using a fixed-effect model showed a statistically significant proportion of patients who received NAVA (217/254) weaned successfully, compared with patients who received other partial support modes (202/258) (OR = 1.93; 95% CI 1.12 to 3.33; *P* = 0.02) (Fig. 2). Subgroup analysis was performed to compare the efficiency of NAVA with the weaning categories, and the result showed a statistical difference favoring NAVA (Additional file 1: Fig. S3). Among 4 studies including 129 patients who were identified as difficult weaning, there was a significant difference between the patients who underwent NAVA versus other weaning modes, with low heterogeneity (I^2^ = 0%) (OR 2.31, 95% CI 1.13 to 4.73, *P* = 0.02).

The certainty of evidence, using the GRADE approach, was moderate (Additional file 1: Table S2). Visual inspection of a funnel plot on weaning success was evaluated and did not suggest the evidence of publication bias (Additional file 1: Fig. S5). TSA suggested the required information size was not reached, but the fact that cumulative z-curve crossed the conventional boundary showed patients undergoing NAVA was more likely to result in successful weaning (Additional file: Fig. S5).

### Secondary outcomes

#### Duration of MV

**Fig. 3:**
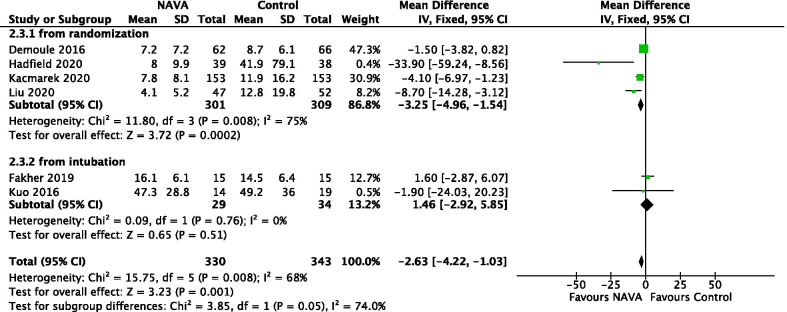
Forest plot for duration of MV from time of intubation. NAVA, neurally adjusted ventilatory assist; PSV, pressure support ventilation, CI, confidence interval

In 6 studies reporting about 673 patients, 4 studies included the duration of MV from randomization [33–36] and 2 studies included the duration of MV from intubation [31, 37]. For all 6 studies, we found a statistically significant probability of lower duration of MV supporting patients undergoing NAVA comparing to other partial support modes (MD = − 2.63; 95% CI − 4.22 to − 1.03; *P* = 0.001), and the heterogeneity was moderate with *I*^2^ = 68% (Fig. 3). Furthermore, subgroup analysis showed a statistically significant difference favoring NAVA in duration of MV when described from randomization. The certainty of evidence was moderate due to inconsistency (Additional file 1: Table S2).

#### Ventilator-free days at day 28

**Fig. 4:**
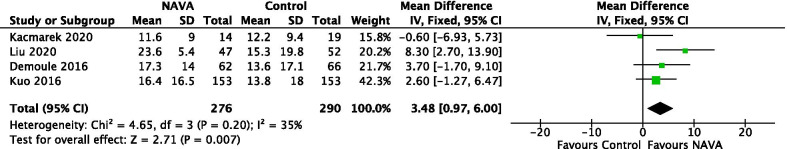
Forest plot for ventilator-free days at day 28. NAVA, neurally adjusted ventilatory assist

For the 4 studies recruiting 566 patients [34–37], patients undergoing NAVA had a greater VFDs compared with patients undergoing other partial support modes (MD = 3.48; 95% CI 0.97 to 6.00; *P* = 0.007) (Fig. 4). The certainty of evidence was moderate due to imprecision (Additional file 1: Table S2).

#### Hospital mortality

**Fig. 5:**
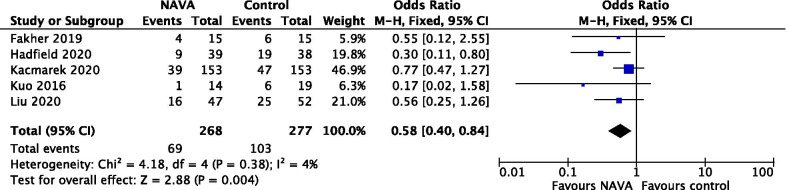
Forest plot for hospital mortality. *NAVA* neurally adjusted ventilatory assist; CI, confidence interval

Hospital mortality was evaluated in 5 studies involving 555 patients [31, 33–35, 37], and the result demonstrated patients ventilated with NAVA had lower hospital mortality compared to patients ventilated with other partial support modes (OR = 0.58; 95% CI 0.40 to 0.84; *P* = 0.004) (Fig. 5). The certainty of evidence was moderate due to imprecision (Additional file 1: Table S2).

#### Other secondary outcomes

There was no statistically significant difference in ICU mortality, hospital LOS, tracheostomy, or adverse events (ventilator-associated pneumonia (VAP), pneumothorax) (Additional file 1: Fig. S6-S9). The certainty of evidence was moderate due to imprecision (Additional file 1: Table S2).

## Discussion

This systematic review identified 7 studies to evaluate the efficacy of NAVA as a weaning mode in intubated patients with previous respiratory failure in terms of the rate of weaning success and other clinically relevant outcomes. The main finding was that, compared with other partial support modes (especially PSV), NAVA was associated with a higher chance of weaning success, lower duration of MV from time of intubation, greater amount of VFDs, and lower hospital mortality. Subgroup analysis suggested that compared with studies involving patients with mixed weaning categories, the benefits of NAVA in terms of weaning success were greater in studies involving patients with difficult weaning.

This systematic review and meta-analysis is the first to evaluate the weaning success in patients undergoing NAVA. Previous physiologic studies have confirmed that NAVA improves patient–ventilator interaction and decreases the occurrence of ventilator under-assist and over-assist [12, 16, 17, 38]. Considering that PVA, a common occurrence in critically ill patients with MV, increases the likelihood of respiratory muscle injury and may be associated with delayed weaning off MV [39], NAVA may be associated with more efficient weaning and has the potential to improve clinical outcomes. However, there is limited evidence on the clinically relevant outcomes for NAVA. Our study demonstrates a biologically plausible association between NAVA and a higher rate of weaning success in intubated patients. However, the efficacy of NAVA in patients with different diseases (chronic obstructive pulmonary disease, acute respiratory distress syndrome (ARDS), neuromuscular diseases) was not evaluated in terms of granular data. Additional studies are further needed.

NAVA and proportional assist ventilation (PAV) are both proportional modes of ventilation readily available in clinical practice and have advantages in reducing PVA [12] and providing the potential for both lung and diaphragm-protective ventilation [40]. Regarding proportional modes of ventilation (either NAVA or PAV, or both), a meta-analysis of 15 studies has shown that proportional modes, when compared with PSV, were associated with a reduction in weaning failure and duration of MV. Reduced duration of mechanical ventilation was found with PAV but not with NAVA [41]. However, our results showed that NAVA was associated with a higher rate of weaning success and lower duration of MV. The potential mechanisms for this effect were speculated as follows: First, PVA is common during MV but underdiagnosed. Previous studies have suggested PVA might lead to lung and vascular injury and thereby was associated with poor clinical outcomes including prolongation of mechanical ventilation [13], increased mortality [42], ICU and hospital stay [43], discomfort [44], and sleep disorders [45]. Recently, most physiological studies have demonstrated that NAVA is associated with a lower asynchrony index (AI) and lower severe asynchrony compared to PSV [12, 45, 46]. Therefore, NAVA may potentially result in weaning success on account of reducing the occurrence of PVA. Second, diaphragm function is a crucial determinant of patients’ outcomes. Mounting evidence demonstrates that ventilator-induced diaphragm dysfunction (VIDD) is significantly associated with difficult weaning and poor clinical outcomes [47–49]. NAVA achieves assisted ventilation by monitoring the electrical activity of diaphragm and reduces VIDD [12, 50]. Worth mentioning, NAVA can prevent over-assistance with the fact that Edi will not disappear completely at high levels of assist because it is required to run the ventilator. NAVA may, therefore, facilitate weaning from MV by preventing or reversing diaphragmatic atrophy [50]. Third, NAVA is considered to reproduce the variability of the neural respiratory drive [40]. Thus, preservation of respiratory variability during NAVA is another potential mechanism for lung protective ventilation and may be an explanation for our results. Fourth, preferential lung recruitment in ARDS is one features of NAVA. Widing et al. [18] found NAVA could decrease recruitment/derecruitment in the ARDS model. [Recruitment/derecruitment can lead to ventilator-induced lung injury (VILI).] The study conducted by Blankman et al. [51] to identify the effect of varying levels of assist during PSV and NAVA showed that NAVA had a better promoting effect on the ventilation of the dependent lung region and less over-assistance. Hence, it could be suggested that NAVA shows beneficial effects on weaning by reducing VILI. Fifth, sleep quality may play an important role in the weaning success. Sleep deprivation may be a risk factor for weaning failure due to decreased respiratory muscle endurance and increased incidence of delirium [6, 43]. Furthermore, previous studies found that weaning time was longer in patients with sleep loss [52]. In the Delisle et al. study, the benefit of improved sleep quality was even more pronounced in NAVA than PSV [20]. Sixth, NAVA has the benefit of using lower sedative doses in acute respiratory failure [21]. Evidence suggests that sedation worsens outcomes in critically ill patients on mechanical ventilation [53]. In addition, long-term sedation may delay liberation from MV and increase the risk of delirium [54, 55].

The differences of underlying weaning status may affect the role of NAVA on weaning success. Therefore, we performed a subgroup analysis in terms of the difficulty and length of the weaning process, namely simple weaning, difficult weaning, and prolonged weaning [6]. In our analysis, two studies included patients with difficult weaning, and two studies included patients with mixed weaning categories. Our results showed that NAVA was associated with higher rate of weaning success in patients with difficult weaning. Therefore, it is speculated that NAVA may have a beneficial effect on patients in which the quality of patient–ventilator interactions is essential, such as patients with difficult weaning. Also, the ventilator allowed monitoring of the diaphragmatic electrical activity in NAVA. So, this group of patients may have a higher rate of successful weaning because of less VIDD. (By monitoring Edi, it is possible to ensure appropriate levels of diaphragm activity and avoids disuse atrophy.)

This meta-analysis also demonstrated that NAVA was associated with a shorter duration of MV and greater VFDs at day 28 compared with other partial support modes. The finding is important as these may facilitate patients’ liberation from MV and discharge from hospital. Previous studies have suggested that severe PVA was associated with worsened outcomes [11, 12, 17]. Therefore, NAVA is speculated to improve these clinically relevant outcomes by reducing PVA, but there is limited evidence to confirm this. In one meta-analysis performed by Chen et al., patients undergoing NAVA had a shorter duration of ventilation than PSV [56]. But in their manuscript, only two studies were included, and the duration of ventilation was not defined clearly.

Our findings also demonstrated a significant association with lower hospital mortality but not ICU mortality and hospital LOS with the use of NAVA compared with other partial support modes. Although it is possible that a higher weaning success may reduce all-cause mortality and shorten the hospital LOS, few data have confirmed this assumption. Potential explanations for these findings may include concomitant complications and different causes of acute respiratory failure among the included patients. Our study also indicated that no significant differences were observed between NAVA and other partial support modes on reduced adverse events (VAP, pneumothorax) and tracheostomy. The possible explanation for the results could be very limited data in included studies. So, larger RCTs are needed to confirm these results.

Our meta-analysis suggests the beneficial effect of NAVA as an alternative to wean, and as well, that NAVA may be associated with lower duration of MV from time of randomization, greater VFDs and lower hospital mortality. The results could be expected given the physiological design of the NAVA mode. It is worth noting that NAVA does have limitations. For example, it is not possible to blind the NAVA arm in studies, since the ventilator screen needs to be viewed, and the patient has an Edi catheter. Also, setting the NAVA level is not a “one size fits all” and is patient-specific. Moreover, NAVA may be associated with a higher prevalence of double triggering in several conditions [12, 16]. Therefore, the above should be kept in mind when interpreting our results.

There are several limitations to our meta-analysis. First, the lack of a detailed weaning protocol implementation in included studies is a major source of heterogeneity which may affect our results. In addition, the included studies involved heterogeneous populations and used variable definitions of outcomes (e.g., duration of MV) despite attempts to reduce clinical heterogeneity. Second, one of the included studies is a crossover trial, which is a theoretical risk that the efficacy of NAVA may be overestimated or underestimated compared with that of other partial support modes. Third, all studies in our analysis had a high risk of performance bias because of the inability to blind the investigators to the method of weaning. So, it is possible that the investigators’ decisions and actions may be influenced, resulting in biased estimates of results. Fourth, not all the data on the tracheostomized patients with successful weaning were included. This may affect the results for the primary outcome in our study. Fifth, five included studies are characterized as small studies of which the sample size was less than 100 patients. Hence, the study effect bias may exist in our results. Further RCTs should be performed to confirm our results.

## Conclusions

Ventilation with the NAVA mode may improve the rate of weaning success compared to other partial support modes for difficult to wean patients. The evaluation of duration of MV, ventilator-free days at day 28, hospital mortality, and successful extubation were in favor of NAVA.

## Supplementary Information

**Additional file 1.** The main results of search strategy, GRADE evidence profile, risk of bias, reporting bias, TSA, subgroup analysis, and secondary outcomes.

## Data Availability

All data generated or analyzed during this study are included in this published article and its supplementary information files.

## Abbreviations

NAVA: Neurally adjusted ventilatory assist
PVA: Patient–ventilator asynchrony
MV: Mechanical ventilation
PSV: Pressure support ventilation
ICU: Intensive care unit
ARDS: Acute respiratory distress syndrome
VIDD: Ventilator-induced diaphragm
VILI: Ventilator-induced lung injury
Edi: Electrical activity of the diaphragm
RCTs: Randomized controlled trials
PRVC: Pressure-regulated volume control
VAP: Ventilator-associated pneumonia
LOS: Length of hospital stay
VFDs: Ventilator-free days
MD: Mean difference
CI: Confidence interval
