# Supplementary material for: Neurally adjusted ventilatory assist as a weaning mode for adults with invasive mechanical ventilation: a systematic review and meta-analysis
Source: Crit Care. 2021 Jun 29;25:222. doi: 10.1186/s13054-021-03644-z (PMC8240429; doi:10.1186/s13054-021-03644-z)
Supplement: Supplementary file 1 — Additional file 1. The main results of search strategy, GRADE evidence profile, risk of bias, reporting bias, TSA, subgroup analysis, and secondary outcomes. [file 13054_2021_3644_MOESM1_ESM.docx]

**Neurally adjusted ventilatory assist as a weaning mode for adults with invasive mechanical ventilation: a systematic review and meta-analysis**

Xueyan Yuan^1^, Xinxing Lu^1^, Yali Chao^1^, Jennifer Beck^2 4 5^, Christer Sinderby^3 4 5^, Jianfeng Xie^1^, Yi Yang^1^, Haibo Qiu^1*^, Ling Liu^1*^

**Additional files 1**

**Table S1 – Search strategy**

**Database: PubMed**

"interactive ventilatory support"[Title/Abstract] OR (("Support"[All Fields] OR. "support. s"[All Fields] OR "supported"[All Fields] OR "supporter"[All Fields] OR "supporter s"[All Fields] OR "supporters"[All Fields] OR "supporting"[All Fields] OR "supportive"[All Fields] OR "supportiveness"[All Fields] OR "supports"[All Fields]) AND "interactive ventilatory"[Title/Abstract]) OR ("Ventilatory"[All Fields] AND "support interactive"[Title/Abstract]) OR "neurally adjusted ventilatory assist"[Title/Abstract] OR "proportional assist ventilation"[Title/Abstract] OR (("assistances"[All Fields] OR "assistant s"[All Fields] OR "assistants"[All Fields] OR "assisted"[All Fields] OR "assisting"[All Fields] OR "assistive"[All Fields] OR "dental assistants"[MeSH Terms] OR ("dental"[All Fields] AND "assistants"[All Fields]) OR "dental assistants"[All Fields] OR "assistant"[All Fields] OR "helping behavior"[MeSH Terms] OR ("helping"[All Fields] AND "behavior"[All Fields]) OR "helping behavior"[All Fields] OR "Assist"[All Fields] OR "assistance"[All Fields] OR "assists"[All Fields]) AND "ventilation proportional"[Title/Abstract]) OR "ventilation proportional assist"[Title/Abstract]

**Database: Embase**

- 1. 'interactive ventilatory support':ab,ti
  2. 'support, interactive ventilatory':ab,ti
  3. 'ventilatory support, interactive':ab,ti
  4. 'neurally adjusted ventilatory assist':ab,ti
  5. 'proportional assist ventilation':ab,ti
  6. 'assist ventilation, proportional':ab,ti
  7. 'ventilation, proportional assist':ab,ti
  8. 1 or 2 or 3 or 4 or 5 or 6 or 7

**Database: Ovid MEDLINE(R) ALL <1946 to November 18, 2018>**

- 1. # 1 neurally adjusted ventilatory assist.mp. or Interactive Ventilatory Support/
  2. # 2 limit 1 to yr="2007 - 2021"

**Database: Cochrane Library**

- 1. Ventilation, Proportional Assist. ti,ab,kw
  2. Support, Interactive Ventilatory. ti,ab,kw
  3. Support, Interactive Ventilatory. ti,ab,kw
  4. Neurally Adjusted Ventilatory Assist. ti,ab,kw
  5. Proportional Assist Ventilation. ti,ab,kw
  6. Assist Ventilation, Proportional. ti,ab,kw
  7. Ventilation, Proportional Assist. ti,ab,kw
  8. (1 or 2 or 3 or 4 or 5 or 6 or 7) and in Trials

**Table S2 GRADE evidence profile for the studies in the meta-analysis**

| **Certainty assessment** | | | | | | | **№ of patients** | | **Effect** | | **Certainty** | **Importance** |
| --- | --- | --- | --- | --- | --- | --- | --- | --- | --- | --- | --- | --- |
| **№ of studies** | **Study design** | **Risk of bias** | **Inconsistency** | **Indirectness** | **Imprecision** | **Other considerations** | **NAVA** | **Control** | **Relative (95% CI)** | **Absolute (95% CI)** |  |  |
| **weaning success-all trials** | | | | | | | | | | | | |
| 4 | randomised trials | serious ^a^ | not serious | not serious | not serious | none | 217/254 (85.4%) | 202/258 (78.3%) | **OR** 1.93 (1.12 to 3.33) | **91 more per 1,000** (from 19 more to 140 more) | ⨁⨁⨁◯ MODERATE | CRITICAL |
| **Weaning success- difficult weaning** | | | | | | | | | | | | |
| 2 | randomised trials | not serious | not serious | not serious | serious ^b^ | none | 41/62 (66.1%) | 31/67 (46.3%) | **OR 2.31** (1.13 to 4.73) | **203 more per 1,000** (from 30 more to 340 more) | ⨁⨁⨁◯ MODERATE | CRITICAL |
| **Duration of MV from time of intubation- all trials** | | | | | | | | | | | | |
| 6 | randomised trials | not serious | serious ^c^ | not serious | not serious | none | 330 | 343 | - | mean **3.88 lower** (7.49 lower to 0.27 lower) | ⨁⨁⨁◯ MODERATE | CRITICAL |
| **Ventilator-free days at day 28- all trials** | | | | | | | | | | | | |
| 4 | randomised trials | not serious | not serious | not serious | serious ^b^ | none | 276 | 290 | - | mean **3.48 higher** (0.97 higher to 6 higher) | ⨁⨁⨁◯ MODERATE | CRITICAL |
| **Hospital mortality- all trials** | | | | | | | | | | | | |
| 5 | randomised trials | not serious | not serious | not serious | serious ^b^ | none | 69/268 (25.7%) | 103/277 (37.2%) | **OR 0.58** (0.40 to 0.84) | **116 fewer per 1,000** (from 180 fewer to 40 fewer) | ⨁⨁⨁◯ MODERATE | CRITICAL |
| **ICU hospital- all trials** | | | | | | | | | | | | |
| 6 | randomised trials | not serious | not serious | not serious | serious ^d^ | none | 61/330 (18.5%) | 79/343 (23.0%) | **OR 0.75** (0.51 to 1.09) | **47 fewer per 1,000** (from 98 fewer to 16 more) | ⨁⨁⨁◯ MODERATE | IMPORTANT |
| **Hospital LOS- all trials** | | | | | | | | | | | | |
| 5 | randomised trials | not serious | not serious | not serious | serious ^d^ | none | 177 | 190 | - | MD **0.91 higher** (2.12 lower to 3.94 higher) | ⨁⨁⨁◯ MODERATE | IMPORTANT |
| **Tracheostomy- all trials** | | | | | | | | | | | | |
| 3 | randomised trials | not serious | not serious | not serious | serious ^d^ | none | 55/239 (23.0%) | 64/243 (26.3%) | **OR 0.88** (0.64 to 1.20) | **24 fewer per 1,000** (from 77 fewer to 37 more) | ⨁⨁⨁◯ MODERATE | IMPORTANT |
| **Adverse events- VAP** | | | | | | | | | | | | |
| 3 | randomised trials | not serious | not serious | not serious | serious ^d^ | none | 19/148 (12.8%) | 15/156 (9.6%) | **OR 1.36** (0.64 to 2.91) | **30 more per 1,000** (from 32 fewer to 140 more) | ⨁⨁⨁◯ MODERATE | IMPORTANT |
| **Adverse events- pneumothorax** | | | | | | | | | | | | |
| 2 | randomised trials | not serious | not serious | not serious | serious ^d^ | none | 4/192 (2.1%) | 4/191 (2.1%) | **OR 0.99** (0.27 to 3.72) | **0 fewer per 1,000** (from 15 fewer to 53 more) | ⨁⨁⨁◯ MODERATE | IMPORTANT |

**CI:** Confidence interval; **OR:** Odds ratio; **MD:** Mean difference

#### Explanations

a. Carryover effects in crossover trial (Ferreira et al)

b. Below optimal information size

c. I2=65%, the heterogeneity was moderate

d. Wide conﬁdence interval including beneﬁts and harms

**Fig. S1 Risk of bias graph**


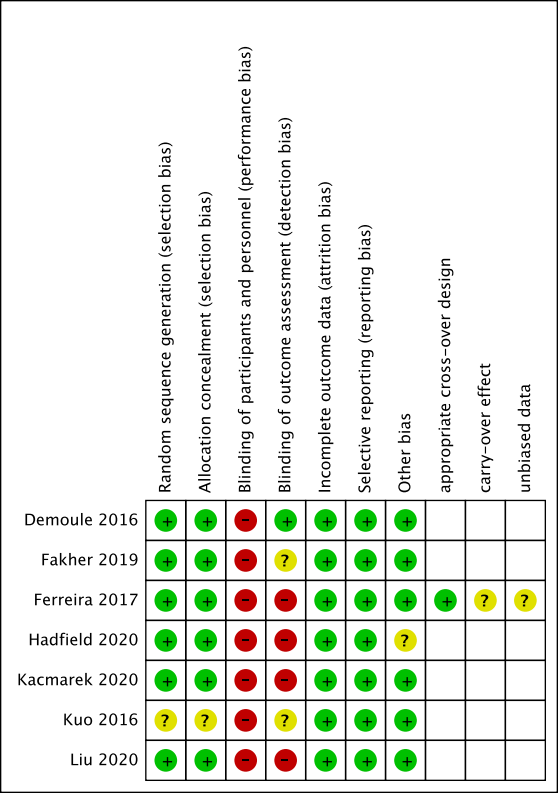


**Fig. S2 Risk of bias summary**


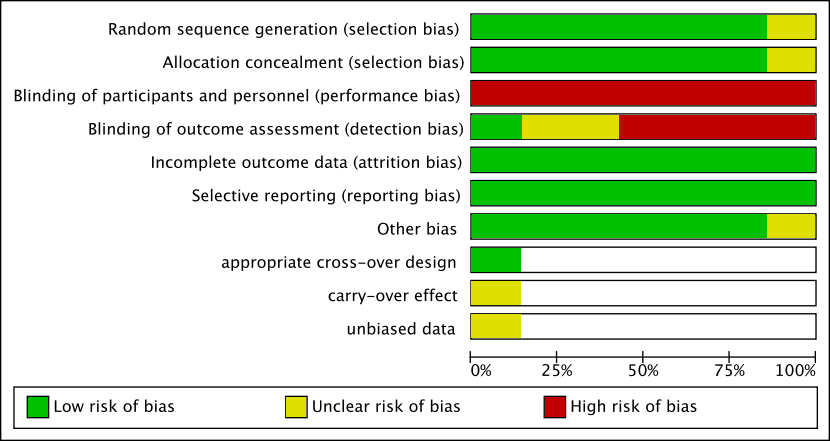


**Fig. S3 Subgroup analysis of weaning success between the two groups with regard to differences weaning categories (difficult weaning vs mix weaning)**

**Fig. S4 Funnel plot for weaning success**

**Fig. S5 Trial sequential analysis of weaning success**

**Fig. S6 Forest plot for ICU mortality**

**Fig. S7** **Forest plot for hospital LOS**

**Fig. S8** **Forest plot for tracheostomy**

**Fig. S9** **Forest plot for adverse events (VAP and pneumothorax)**
